# Supplementary material for: Analysis of Comparative Transcriptome and Positively Selected Genes Reveal Adaptive Evolution in Leaf-Less and Root-Less Whisk Ferns
Source: Plants (Basel). 2022 Apr 28;11(9):1198. doi: 10.3390/plants11091198 (PMC9103481; doi:10.3390/plants11091198)
Supplement: Supplementary file 1 [file plants-11-01198-s001.zip › Supplementary Figure S1-S3.pdf]

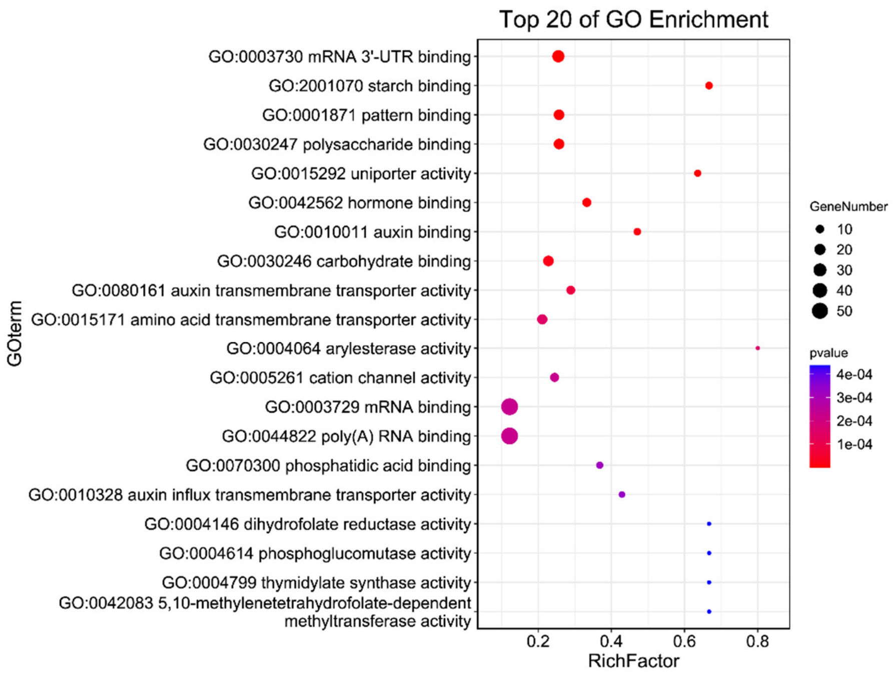

Figure S1. The GO enrichment of P. nudum specific gene families.

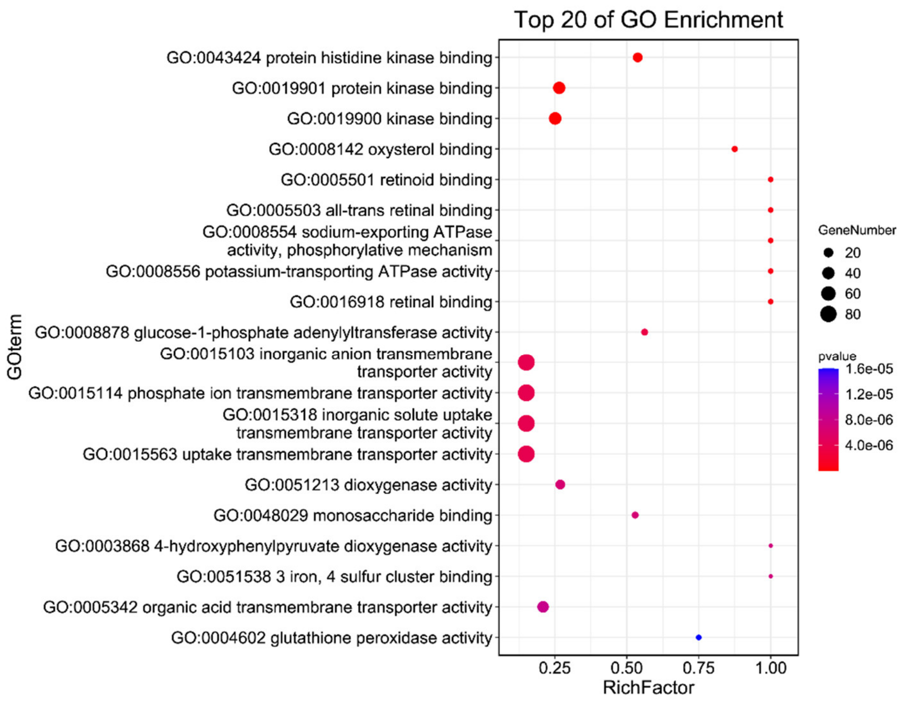

Figure S2. The GO enrichment of T. tannensis specific gene families.

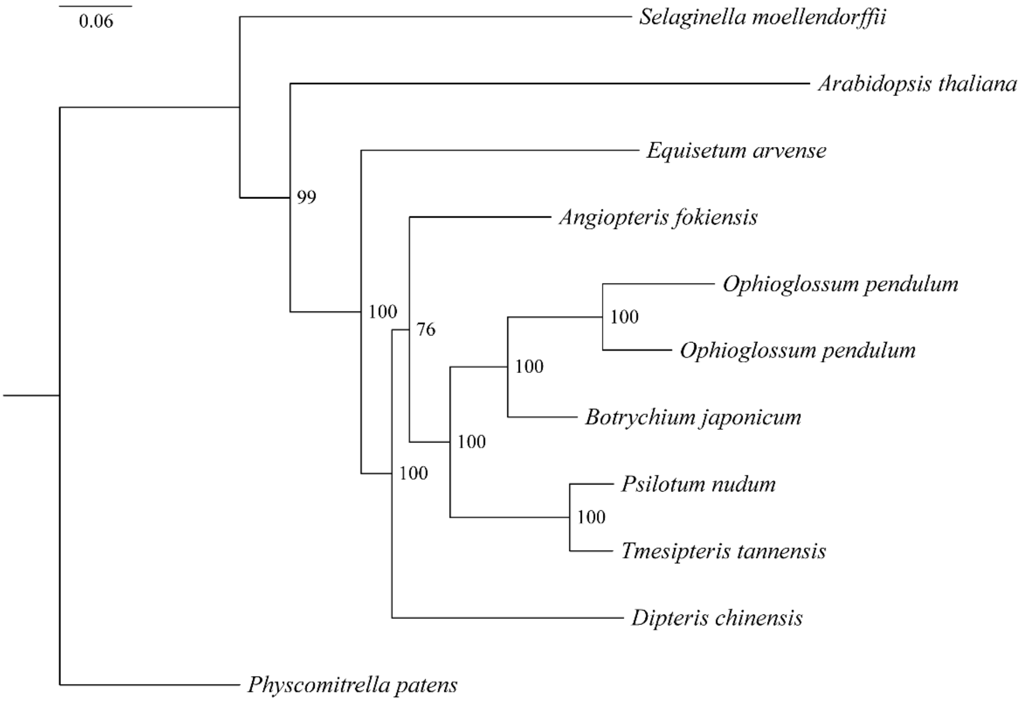

Figure S3. Phylogenetic tree constructed from single-copy orthogroups. Evolutionary relationship reconstructed from concatenated alignment of the amino acid sequences of 65 single-copy orthogroups. Bootstrap values are labeled at the nodes, and the branch lengths unit are depicted in the top left corner.
